# Using Artificial Intelligence ChatGPT to Access Medical Information About Chemical Eye Injuries: Comparative Study

**DOI:** 10.2196/73642

**Published:** 2025-08-13

**Authors:** Layan Yousef Alharbi, Rema Rashed Alrashoud, Bader Shabib Alotaibi, Abdulaziz Meshal Al Dera, Raghad Saleh Alajlan, Reem Rashed AlHuthail, Dalal Ibrahim Alessa

**Affiliations:** 1 College of Medicine, Imam Mohammad Ibn Saud Islamic University (IMSIU), Prince Mohammed Ibn Salman Ibn Abdulaziz Road, Riyadh, 13318, Saudi Arabia, 966 532816087; 2 Department of Ophthalmology, College Of Medicine, Imam Mohammad Ibn Saud Islamic University (IMSIU), Riyadh, Saudi Arabia

**Keywords:** artificial intelligence, ChatGPT, chemical eye injuries, ophthalmology, medical information, patient education, ICD-9, ICD-10, readability

## Abstract

**Background:**

Chemical ocular injuries are a major public health issue. They cause eye damage from harmful chemicals and can lead to severe vision loss or blindness if not treated promptly and effectively. Although medical knowledge has advanced, accessing reliable and understandable information on these injuries remains a challenge. This is due to unverified web-based content and complex terminology. Artificial intelligence tools like ChatGPT provide a promising solution by simplifying medical information and making it more accessible to the general public.

**Objective:**

This study aims to assess the use of ChatGPT in providing reliable, accurate, and accessible medical information on chemical ocular injuries. It evaluates the correctness, thematic accuracy, and coherence of ChatGPT’s responses compared with established medical guidelines and explores its potential for patient education.

**Methods:**

A total of 9 questions were entered into ChatGPT regarding various aspects of chemical ocular injuries. These included the definition, prevalence, etiology, prevention, symptoms, diagnosis, treatment, follow-up, and complications. The responses provided by ChatGPT were compared with the *International Classification of Diseases-9 *and *International Classification of Diseases-10* guidelines for chemical (alkali and acid) injuries of the conjunctiva and cornea. The evaluation focused on criteria such as correctness, thematic accuracy, and coherence to assess the accuracy of ChatGPT’s responses. The inputs were categorized into 3 distinct groups, and statistical analyses, including Flesch–Kincaid readability tests, ANOVA, and trend analysis, were conducted to assess their readability, complexity, and trends.

**Results:**

The results showed that ChatGPT provided accurate and coherent responses for most questions about chemical ocular injuries, demonstrating thematic relevance. However, the responses sometimes overlooked critical clinical details or guideline-specific elements, such as emphasizing the urgency of care, using precise classification systems, and addressing detailed diagnostic or management protocols. While the answers were generally valid, they occasionally included less relevant or overly generalized information. This reduced their consistency with established medical guidelines. The average Flesch Reading Ease Score was 33.84 (SD 2.97), indicating a fairly challenging reading level, while the Flesch–Kincaid Grade Level averaged 14.21 (SD 0.97), suitable for readers with college-level proficiency. The passive voice was used in 7.22% (SD 5.60%) of sentences, indicating moderate reliance. Statistical analysis showed no significant differences in the Flesch Reading Ease Score (*P*=.38), Flesch–Kincaid Grade Level (*P*=.55), or passive sentence use (*P*=.60) across categories, as determined by one-way ANOVA. Readability remained relatively constant across the 3 categories, as determined by trend analysis.

**Conclusions:**

ChatGPT shows strong potential in providing accurate and relevant information about chemical ocular injuries. However, its language complexity may prevent accessibility for individuals with lower health literacy and sometimes miss critical aspects. Future improvements should focus on enhancing readability, increasing context-specific accuracy, and tailoring responses to a person’s needs and literacy levels.

## Introduction

The internet has become a significant source of health-related information. In the early 2000s, 4.5% of global internet searches were health-related. However, much of the web-based medical information is often inappropriate or even harmful due to unverified content and insufficient internet regulations. Additionally, even when the information is accurate, it may be presented in language too complex for the general public, making it inaccessible [1-3]. Artificial intelligence (AI), a field of computer science, focuses on creating systems capable of tasks traditionally requiring human intelligence, such as reasoning, learning, problem-solving, perception, and language comprehension [4]. In November 2022, OpenAI introduced “ChatGPT,” a large language model trained on extensive multilingual text datasets, enabling it to generate human-like responses to textual inputs. Chatbots, as AI-powered apps, are designed to simulate human conversations effectively through text or voice interactions [5].

With its extensive knowledge base and complex parameters, it allows users to ask questions and receive answers that demonstrate advanced, human-like reasoning [6]. Since its release, ChatGPT has gained significant popularity and is rapidly becoming a common tool for individuals to seek various types of information via the web [7]. Currently, many studies are assessing the capabilities and potential applications of this chatbot, but the reliability of the information provided by ChatGPT still requires validation.

ChatGPT has been recognized for its potential to revolutionize the entire medical field across several areas, such as patient care, health care systems and professionals, research, and education and training [8]. A 2020 survey indicated that 55% of Europeans aged 16‐74 years searched for health-related information on the internet, reflecting a 21% increase since 2010. In the United States, the percentage rose from 62.8% in 2008% to 74.7% in 2017, showing an 11.9% increase [9,10]. Because ChatGPT is still new, little is known about how it will affect society in the future, especially in the medical field. In the future, both patients and health care workers might move from using the internet in a basic way to getting more interactive, AI-supported information. Instead of looking up facts about a disease or treatment, they might ask the AI directly, “How should this disease be treated?” or “What are the complications of this treatment?” Learning how AI works and checking how good its answers are will be a big but necessary job for future health care workers. Mistakes or wrong medical information from AI could have serious and even harmful effects in the worst cases [11].

AI has made significant progress in health care, especially in several areas of ophthalmology [12,13]. Since the complexity of ophthalmology makes it highly challenging for ChatGPT to provide accurate answers to questions in this field, the use of ChatGPT to access medical information in ophthalmology remains unexplored. For this reason, further validation studies are crucial, particularly to evaluate the effectiveness of ChatGPT in addressing a broad spectrum of eye disease–related questions within the clinical practice setting in Saudi Arabia.

Considering the growing trend and increasing popularity of ChatGPT, it is likely that people will rely on it to ask questions related to ophthalmology, particularly in cases of emergency eye injuries such as those caused by chemicals. As a result, we aimed to assess the accuracy of the information provided by ChatGPT in ophthalmology, particularly on chemical injury of the conjunctiva and cornea. Additionally, we sought to evaluate the potential for incorrect or even harmful information that could pose risks to patients using the chatbot independently. This study aims to assess the use of AI ChatGPT for accessing medical information on chemical corneal and conjunctival injuries.

## Methods

### Generating Medical Information

The following data were generated on December 21, 2024, using the official ChatGPT-4-turbo app by OpenAI. To evaluate the ability of ChatGPT to provide accurate medical information, we presented the AI ChatGPT with a series of questions related to chemical ocular injury. These questions covered topics such as the definition, prevalence, etiology, prevention, symptoms, diagnosis, treatment, follow-up, and complications of chemical ocular injury. A total of 9 distinct ChatGPT responses were analyzed, corresponding to the 9 queries submitted.

ChatGPT responses were then compared with the *International Classification of Diseases-9* (ICD-9) and *ICD-10* guidelines for chemical (alkali and acid) injuries of the conjunctiva and cornea. These responses were evaluated based on criteria such as correctness, thematic accuracy, and coherence to assess their accuracy. ChatGPT-4-turbo uses a probabilistic algorithm, meaning it uses random sampling to generate a variety of responses, which may include different answers to the same query. This investigation focused solely on ChatGPT’s initial response to each query, without regenerating answers. Additional clarifications or explanations were not allowed. All queries were entered into a ChatGPT account owned by the author in a single day, ensuring accuracy in grammar and syntax. Each query was placed into a separate dialogue window to eliminate confounding factors and ensure the accuracy and precision of the responses, as ChatGPT-4-turbo can adapt based on the details of each interaction. The study did not require institutional review board approval, as it did not involve human participants, and no patient-identifying information was used.

### Evaluation of Answers

In evaluating the responses to each question, we compared them with established guidelines and assessed them based on their correctness, thematic accuracy, and coherence. The criteria are organized as shown in Textbox 1.

Textbox 1. **Criteria for evaluation of answers.**

Correctness

Correct: The entire answer is correct.

Partially correct: The core statement of the answer is correct, but the rest of the answer contains one or more errors.

Incorrect: The core statement of the answer is incorrect.

Thematic accuracy

Applicable: The answer identifies the central concept and is thematically specific.

Partially applicable: The answer identifies the central concept, but also partially addresses an unrelated topic.

Not applicable: The answer does not identify the central concept or target an unrelated topic.

Coherence

Coherent: The core message of the answer is fully supported by the rest of the answer.

Partially coherent: The core statement of the answer is essentially confirmed by the rest of the answer, but there are deviating statements/contradictions in the rest of the answer.

Incoherent: The core statement of the answer contradicts the rest of the answer [14].

### Data Analysis

We performed an in-depth linguistic analysis to evaluate the readability and complexity of the AI-generated responses. To achieve this, 2 main readability scores, the Flesch Reading Ease Score (FRES) and the Flesch–Kincaid Grade Level (FKGL), were computed using standard formulas. FRES evaluates text complexity on a scale from 0 to 100, where lower scores suggest more difficult reading.


$$FRES=206.835-1.015\times \left(\frac{Words}{Sentences}\right)-84.6\times \left(\frac{Syllables}{Words}\right)$$

FKGL approximates the US education grade level required to comprehend the text.


$$FKGL=0.39\times \left(\frac{Words}{Sentences}\right)+11.8\;\times \left(\frac{Syllables}{Words}\right)-15.59$$

### Sentence Identification and Data Extraction

All textual analyses were performed on the final output for each of the 9 sections (definition, prevalence, causes, preventive measures, symptoms, diagnosis, treatment, follow-up, and complications). Each section’s text was analyzed separately, without alteration, ensuring that the analysis reflected the original language. Sentences were identified by their terminal punctuation (periods, exclamation points, or question marks).

### Passive Voice Identification, Word and Syllable Counting

Words were counted by recognizing sequences of characters separated by whitespace or punctuation. Contractions (eg, “don’t”) were treated as single words. Hyphenated words (eg, “well-labeled”) were similarly counted as single words, ensuring uniform treatment of such constructions. A standard English syllable-counting heuristic was applied to each word, examining vowel groupings and common exceptions (eg, silent “e” or diphthongs). This automated count was then manually reviewed for potential errors, especially in acronyms or chemical abbreviations, to ensure accurate syllable totals. Each sentence was manually inspected to detect passive constructions, characterized by a form of “be” followed by a past participle (eg, “...was performed”). Such sentences were marked as passive. The total count of passive sentences in each section was divided by the total sentence count to calculate the percentage of passive use.

In our study, we first calculated the average (mean) and variability (SD) for both the FRES and FKGL indices. We also calculated the percentage of passive sentences to assess the overall readability performance of ChatGPT. We included the analysis of the percentage of passive sentences because it can impact the readability and comprehension of the text. Passive structures are typically more difficult to read and understand, especially for individuals with lower reading proficiency.

Subsequently, we categorized the 9 questions about chemical ocular injury into three distinct groups: (1) definition, prevalence, and etiology; (2) prevention, symptoms, and diagnosis; and (3) treatment, follow-up, and complications. For each category, we calculated the mean and SD for the FRES, FKGL, and the percentage of passive sentences in ChatGPT responses. These initial steps set the stage for more detailed statistical analyses, including ANOVA and trend analysis, which allowed us to investigate whether the readability performance of ChatGPT varied across the different question categories. ANOVA was used to compare the means of 3 or more groups to determine if there were any statistically significant differences among them. In this study, ANOVA was applied to compare the means of the readability metrics across the 3 categories of questions. Trend analysis was performed to identify patterns and shifts in the readability metrics across the categories. The mean and SD for each readability metric were plotted to visualize trends in the data. This analysis helped uncover any systematic changes in readability and complexity of the responses based on the type of information provided. Hypothesis testing was performed at a 5% significance level. This comprehensive analytical approach enabled us to gain a deeper understanding of the textual characteristics of the AI-generated content and thoroughly assess its suitability for patient education.

### Ethical Considerations

This study did not involve human participants, clinical data, or personal identifiers and was therefore exempt from institutional ethics review. As no human participants were involved, informed consent was not applicable. Additionally, no personal data were collected or analyzed, as all data were generated using the ChatGPT-4-turbo app developed by OpenAI. Since no participants were recruited or compensated, compensation considerations were not applicable. Furthermore, no identifiable images of individuals were included in the manuscript or any supplemental materials.

## Results

### Qualitative Analysis

Figures 1-15 illustrate ChatGPT responses to a series of questions addressing various aspects of chemical ocular injuries, including their definition, prevalence, etiology, prevention, symptoms, diagnosis, treatment, follow-up, and potential complications. The initial objective of this analysis is to evaluate the accuracy of ChatGPT’s answers to each of these questions, ensuring that the information provided aligns with established medical knowledge.

**Figure 1.**
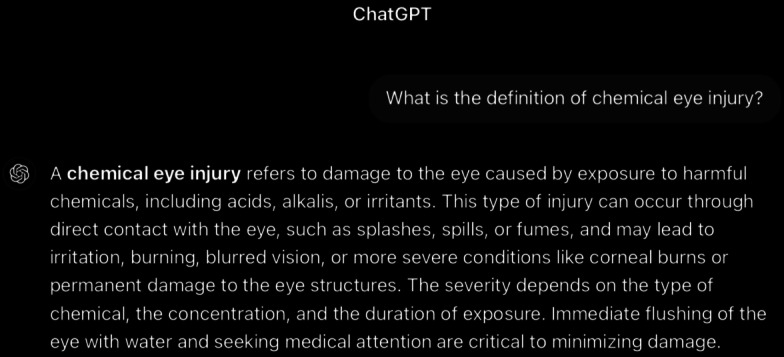
ChatGPT-4-turbo’s response to the question “What is the definition of chemical eye injury?” generated on December 21, 2024, as part of a comparative study assessing artificial intelligence–generated medical information on chemical ocular injuries. The study was conducted in Riyadh, Saudi Arabia.

**Figure 2.**
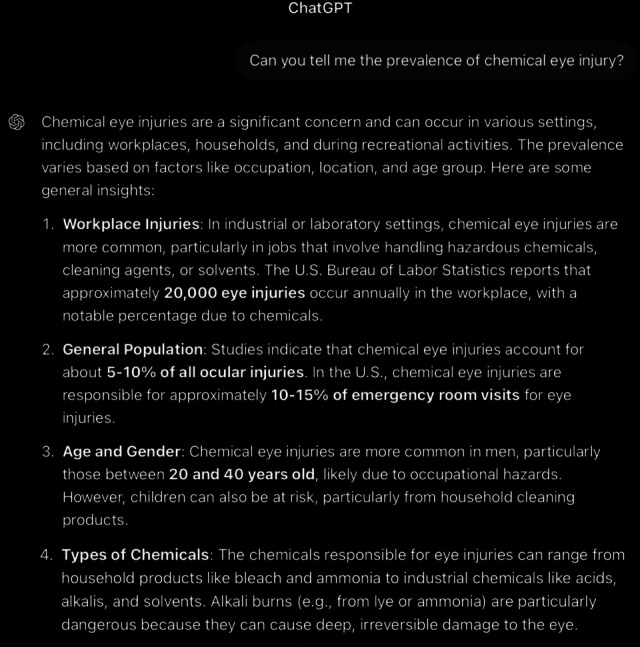
ChatGPT-4-turbo’s response to the question “Can you tell me the prevalence of chemical eye injury?” generated on December 21, 2024, as part of a comparative study assessing artificial intelligence–generated medical information on chemical ocular injuries. The study was conducted in Riyadh, Saudi Arabia.

**Figure 3.**
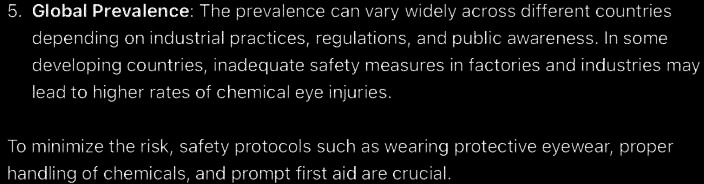
The rest of ChatGPT-4-turbo’s response to the question “can you tell me the prevalence of chemical eye injury?” generated on December 21, 2024, as part of a comparative study assessing artificial intelligence–generated medical information on chemical ocular injuries. The study was conducted in Riyadh, Saudi Arabia.

**Figure 4.**
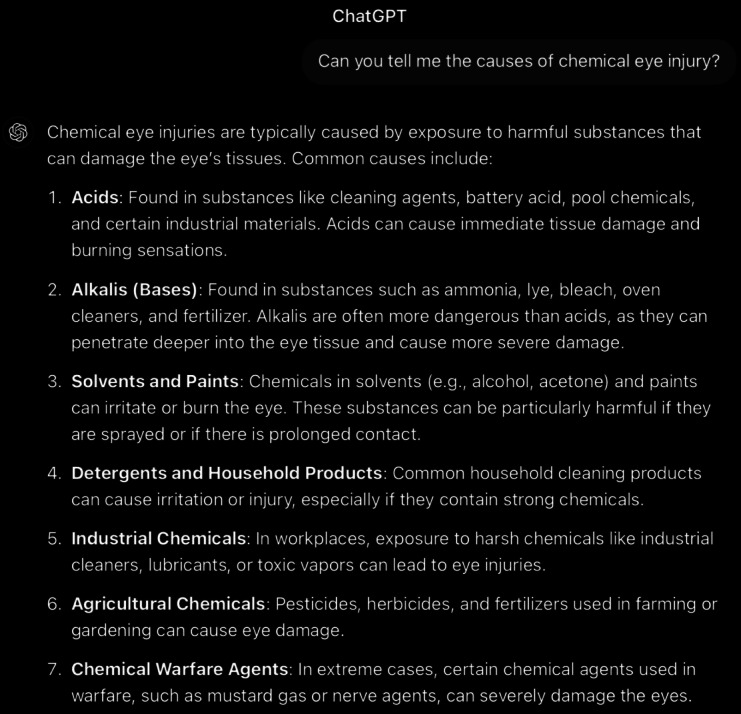
ChatGPT-4-turbo’s response to the question “can you tell me the causes of chemical eye injury” generated on December 21, 2024, as part of a comparative study assessing artificial intelligence–generated medical information on chemical ocular injuries. The study was conducted in Riyadh, Saudi Arabia.

**Figure 5.**
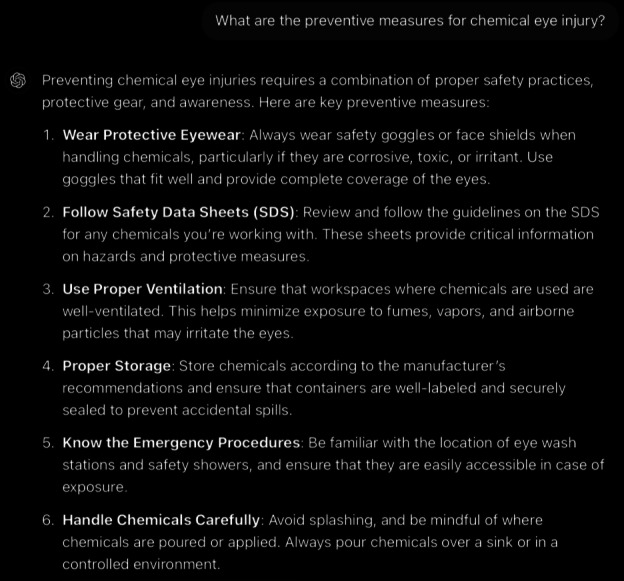
ChatGPT-4-turbo’s response to the question “What are the preventive measures of chemical eye injury?” generated on December 21, 2024, as part of a comparative study assessing artificial intelligence–generated medical information on chemical ocular injuries. The study was conducted in Riyadh, Saudi Arabia.

**Figure 6.**
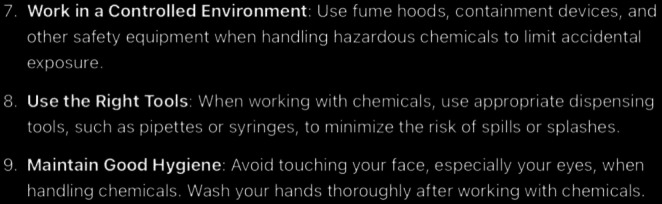
Rest of ChatGPT-4-turbo’s response to the question “What are the preventive measures of chemical eye injury?” generated on December 21, 2024, as part of a comparative study assessing artificial intelligence–generated medical information on chemical ocular injuries. The study was conducted in Riyadh, Saudi Arabia.

**Figure 7.**
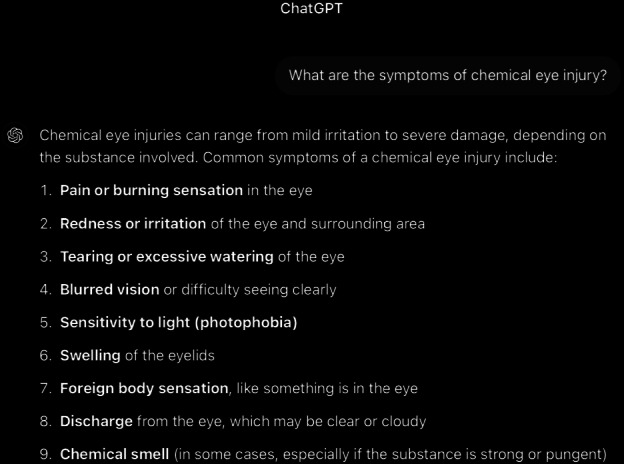
ChatGPT-4-turbo’s response to the question “What are the symptoms of chemical eye injury?” generated on December 21, 2024, as part of a comparative study assessing artificial intelligence–generated medical information on chemical ocular injuries. The study was conducted in Riyadh, Saudi Arabia.

**Figure 8.**
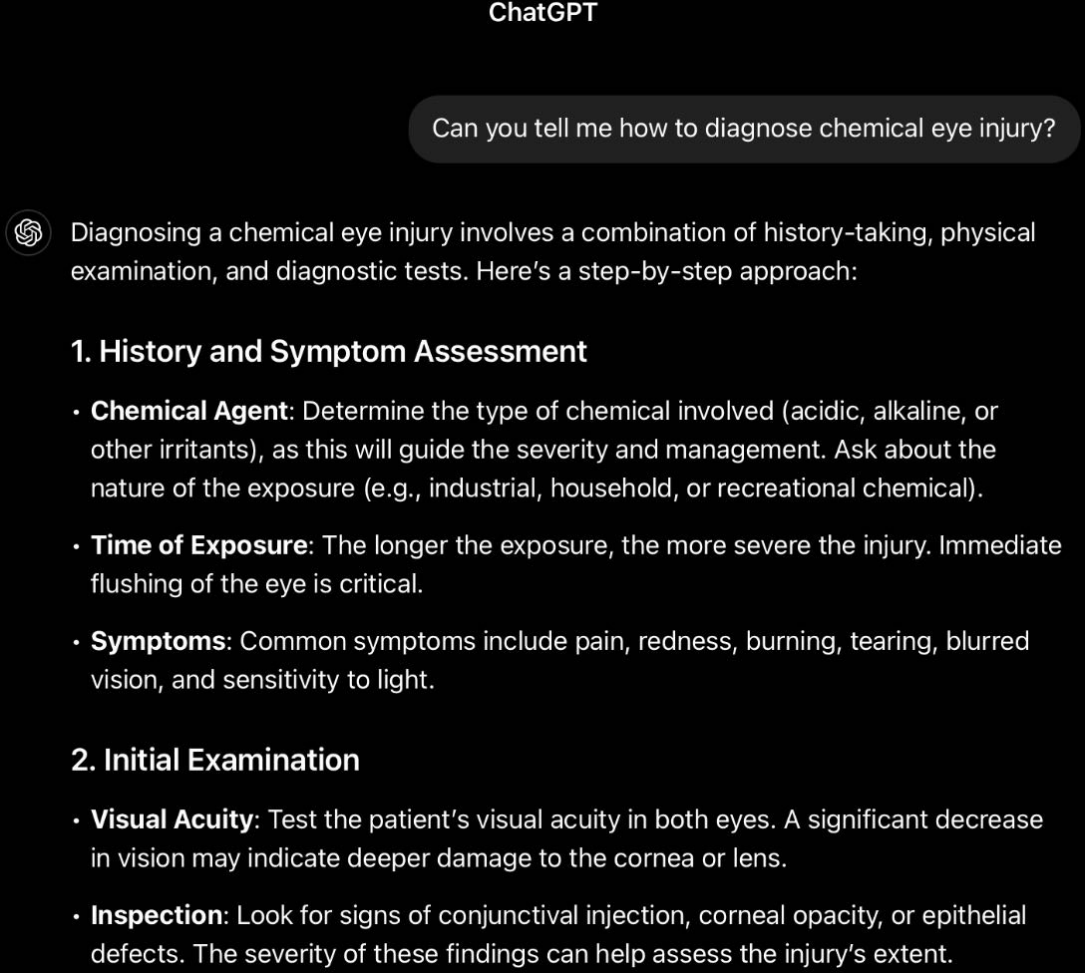
ChatGPT-4-turbo’s response to the question “Can you tell me how to diagnose chemical eye injury?” generated on December 21, 2024, as part of a comparative study assessing artificial intelligence–generated medical information on chemical ocular injuries. The study was conducted in Riyadh, Saudi Arabia.

**Figure 9.**
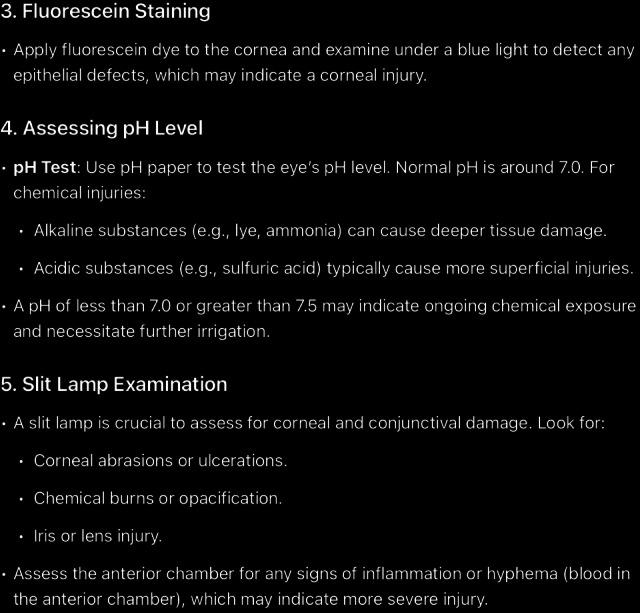
The rest of ChatGPT-4-turbo’s response to the question “can you tell me how to diagnose chemical eye injury?” generated on December 21, 2024, as part of a comparative study assessing artificial intelligence–generated medical information on chemical ocular injuries. The study was conducted in Riyadh, Saudi Arabia.

**Figure 10.**
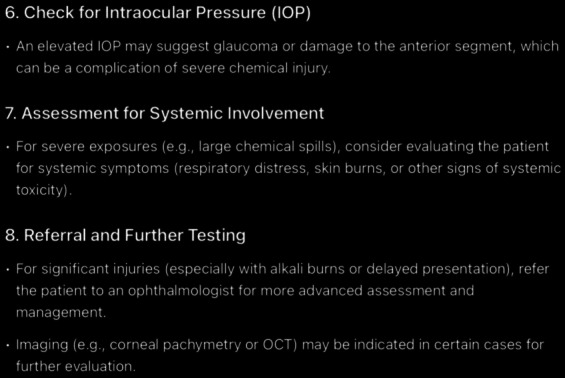
The rest of ChatGPT-4-turbo’s response to the question “Can you tell me how to diagnose chemical eye injury?” generated on December 21, 2024, as part of a comparative study assessing artificial intelligence–generated medical information on chemical ocular injuries. The study was conducted in Riyadh, Saudi Arabia.

**Figure 11.**
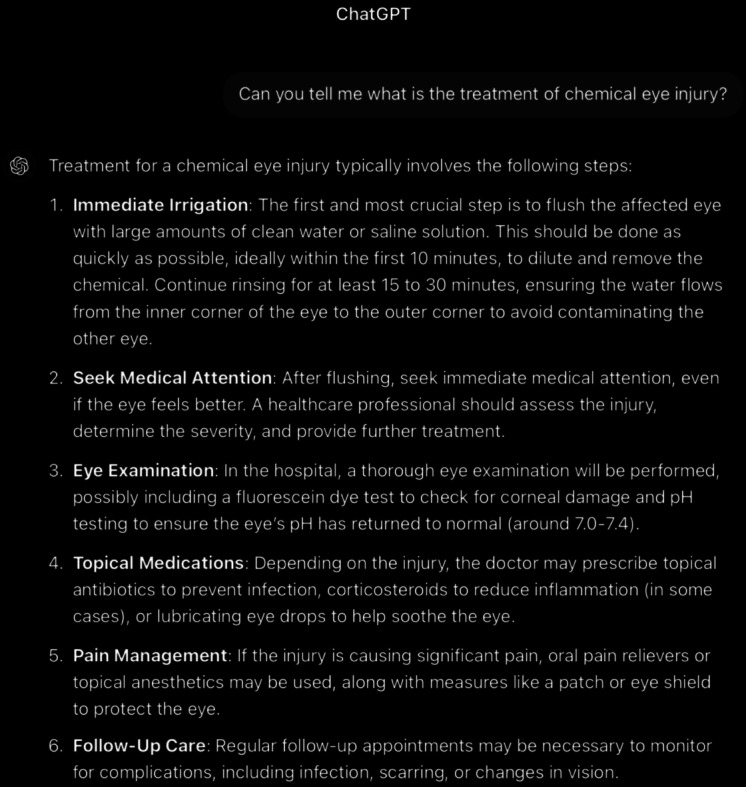
ChatGPT-4-turbo’s response to the question “can you tell me what the treatment of chemical eye injury is?” generated on December 21, 2024, as part of a comparative study assessing artificial intelligence–generated medical information on chemical ocular injuries. The study was conducted in Riyadh, Saudi Arabia.

**Figure 12.**
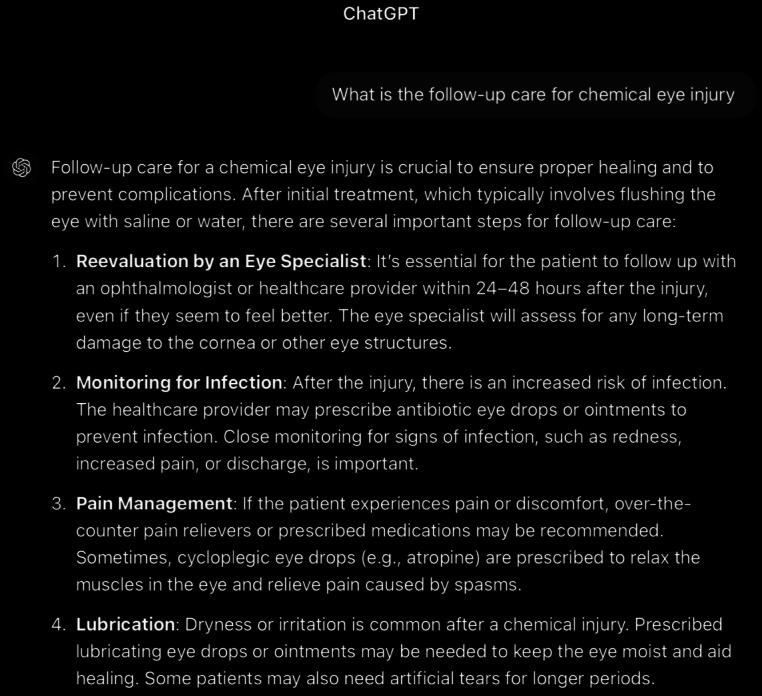
ChatGPT-4-turbo’s response to the question “What is the follow-up care for chemical eye injury” generated on December 21, 2024, as part of a comparative study assessing artificial intelligence–generated medical information on chemical ocular injuries. The study was conducted in Riyadh, Saudi Arabia.

**Figure 13.**
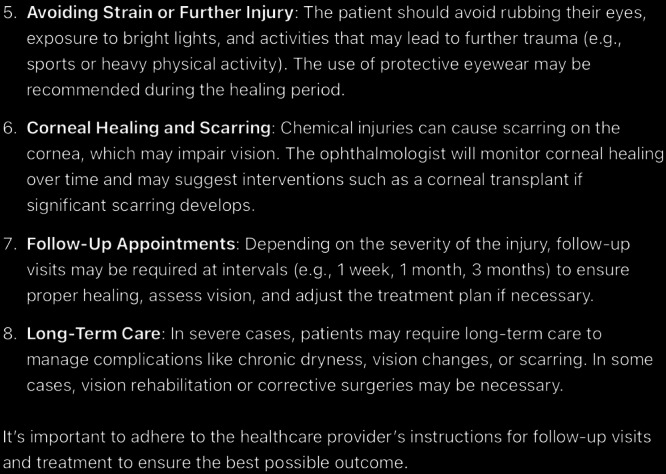
The rest of ChatGPT-4-turbo’s response to the question “What is the follow-up care for chemical eye injury” generated on December 21, 2024, as part of a comparative study assessing artificial intelligence–generated medical information on chemical ocular injuries. The study was conducted in Riyadh, Saudi Arabia.

**Figure 14.**
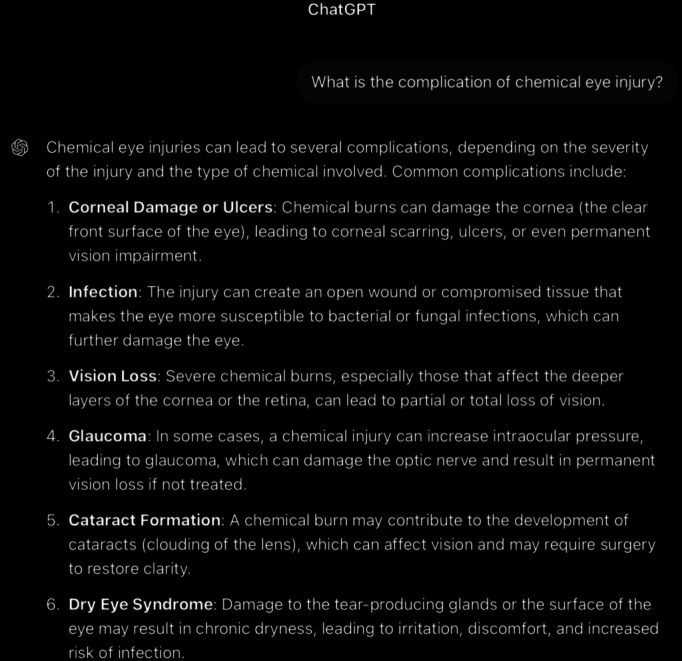
ChatGPT-4-turbo’s response to the question “what is the complication of chemical eye injury” generated on December 21, 2024, as part of a comparative study assessing artificial intelligence–generated medical information on chemical ocular injuries. The study was conducted in Riyadh, Saudi Arabia.

**Figure 15.**
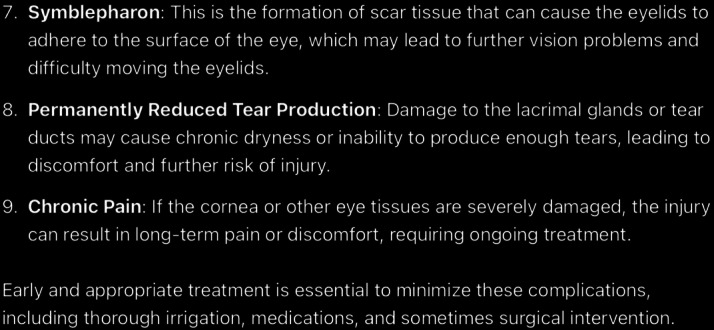
The rest of ChatGPT-4-turbo’s response to the question “what is the complication of chemical eye injury” generated on December 21, 2024, as part of a comparative study assessing artificial intelligence–generated medical information on chemical ocular injuries. The study was conducted in Riyadh, Saudi Arabia.

For the definition question (Figure 1), ChatGPT defines chemical eye injury as it is commonly defined in the medical literature. The analysis of ChatGPT’s response shows that it is correct, providing an accurate description of chemical eye injuries, including their causes, symptoms, and some complications. It also briefly notes the importance of immediate treatment and the factors that determine the severity. It is thematically accurate, as it identifies the central concept and remains fully focused on the topic. The response is also coherent, with its core statement supported by the rest of the answer without contradictions. In comparison, ChatGPT’s response offers a broader explanation, detailing causes (acids, alkalis, and irritants), symptoms (irritation, burning, and blurred vision), and a brief mention of treatment (flushing and medical attention). Meanwhile, the *ICD-9* and *ICD-10 *guidelines are more concise, emphasizing the urgency and severity of chemical eye injuries and targeting medical professionals or individuals needing a quick understanding of the condition.

ChatGPT’s response, while accurate and comprehensive, fails to sufficiently emphasize that chemical eye injuries are true emergencies that demand immediate attention and prompt intervention. It primarily focuses on describing general information, including causes, symptoms, and general treatment. This lack of emphasis on the emergency nature of such injuries may diminish the critical need for quick action.

For the prevalence question (Figures2  3), the analysis of ChatGPT’s response reveals that it is partially correct. It provides accurate and detailed information on the prevalence of chemical eye injuries, including key statistics and contributing factors such as workplace exposure, gender, and age. However, in the general population, ChatGPT’s response states that chemical eye injuries account for about 5%‐10% of all ocular injuries, which is incorrect according to *ICD-9* and *ICD-10* guidelines. These guidelines indicate that chemical injuries to the eye represent between 11.5% and 22.1% of ocular traumas. The response is also partially accurate in terms of thematic accuracy. Although it stays focused on the central topic of the prevalence of chemical eye injuries and addresses relevant subtopics, such as industrial versus domestic settings and global variations, it also discusses the types of chemicals, which was not the main question. Furthermore, it is coherent, with the core statements consistently supported by the detailed explanations provided throughout the response.

In comparison, ChatGPT’s response is more detailed and comprehensive, covering multiple aspects, including workplace statistics (eg, 20,000 injuries annually in the United States), demographic factors (eg, men aged 20‐40 years), and the influence of chemical types. In contrast, the guidelines provide a more concise overview, primarily focusing on general prevalence rates (11.5%‐22.1%), demographic risks (young men and children), and the primary setting of workplace injuries. While both answers are accurate and coherent, ChatGPT’s response offers a broader and more informative perspective by incorporating additional data and global context, whereas the guidelines focus on essential statistics and trends, making it more concise. ChatGPT’s response failed to mention that alkali injuries are more common than acid injuries and are often caused by building materials and cleaning agents, as this exclusion is significant for understanding and managing chemical eye injuries effectively.

For the causes question (Figure 4), the analysis of ChatGPT’s response to the etiology of chemical eye injuries reveals that it is partially correct, as it identifies that chemical injuries can be caused by acids and alkalis. However, some of the listed etiologies are not mentioned in the guidelines, including solvents, detergents, industrial chemicals, agricultural chemicals, and chemical warfare agents. Moreover, the response lacks specificity in its categorization and examples compared with the *ICD-9* and *ICD-10* guidelines. For instance, ChatGPT’s response mentions “cleaning agents, battery acid, and pool chemicals” for acids but does not specify compounds like sulfuric acid (H₂SO₄) or hydrofluoric acid, as outlined in the guidelines. ChatGPT’s response lacks the specific and systematic presentation of substances that the guidelines offer, which is essential for clinical and educational purposes. Thematic accuracy is applicable. The response identifies the central concept (causes of chemical eye injuries) and stays fully focused on the topic. The response is coherent, with its main statement consistently supported by the rest of the answer and free from contradictions.

For the preventive question (Figures5  6), the analysis of ChatGPT’s response shows that it is correct, providing accurate and comprehensive preventive measures that align with standard practices. It emphasizes wearing protective eyewear, following safety protocols, proper chemical handling, and the use of emergency equipment. Thematic accuracy is applicable. The response identifies the central concept (preventing chemical eye injuries) and stays fully focused on the topic. All measures are practical and thematically relevant. The response is coherent, and the answer’s core statement (preventing chemical eye injuries through safety practices) is well-supported throughout. It does not contradict itself and maintains logical progression.

While the response provides detailed preventive strategies, it could more effectively highlight the Occupational Safety and Health Administration regulations mentioned in the guidelines to emphasize compliance with mandatory workplace safety standards. The guidelines specifically mandate the use of protective eyewear in workplace settings, and although the response addresses this and its proper use, it does not sufficiently stress its “mandatory” nature under regulatory requirements. Additionally, the response includes broader prevention measures, such as hygiene practices, proper ventilation, and emergency procedures, which are practical for general audiences but not clearly covered in the guidelines. To improve, the response should emphasize the mandatory nature of protective eyewear per Occupational Safety and Health Administration regulations.

For the symptoms question (Figure 7), the analysis of ChatGPT’s response shows that it is partially correct. The answer includes several accurate and commonly reported symptoms, such as pain and tearing. However, some of the listed symptoms deviate from the most clinically significant findings emphasized in the guidelines. The inclusion of “chemical smell” is not supported by standard guidelines and is not a defining symptom of chemical eye injury. Similarly, “foreign body sensation” may occur, but is not a primary or hallmark symptom. Moreover, the answer neglects “blepharospasm” and “reduced visual acuity”, which are key symptoms in the guidelines. Thematic accuracy is partially applicable. While the answer broadly addresses the concept of chemical eye injury symptoms, it does not focus on the critical symptoms highlighted in the guidelines (severe pain, epiphora, blepharospasm, and reduced visual acuity), and by including less relevant symptoms (eg, “chemical smell”), it partially diverts attention from the more urgent diagnostic features. The response is also partially coherent. The core statement that chemical eye injuries can cause a range of symptoms is supported by the list. However, the inclusion of peripheral symptoms (“chemical smell” and “foreign body sensation”) introduces inconsistencies, as these are not central to the condition and can detract from the urgency of hallmark symptoms.

For the diagnosis question (Figures 8-10), the analysis of ChatGPT’s response shows that it is correct. It accurately describes the diagnostic process for chemical eye injuries. It includes key steps such as history-taking to determine the type of chemical, exposure time, and symptoms, followed by an initial examination of visual acuity and inspection for physical damage, including the use of fluorescein staining, pH testing, slit lamp examination, and intraocular pressure measurement. It is thematically accurate, as it identifies the central concept and remains fully focused on the topic. The response is also coherent, with its core statement supported by the rest of the answer without contradictions.

In comparison, ChatGPT’s response appropriately begins with the importance of obtaining a patient’s history in diagnosing chemical eye injuries. It suggests asking about the chemical agent to identify its type, the nature of exposure (household or industrial), the timing of exposure, and common symptoms such as pain, redness, burning, tearing, blurred vision, and light sensitivity. However, it omits several critical aspects that have been mentioned in *ICD-9* and *ICD-10* guidelines, including assessing the chemical’s toxicity, understanding the depth of ocular penetration, determining the area of involvement, and asking whether the eyes were rinsed afterward and for how long. Additionally, it fails to mention the need to ask about the use of eye protection at the time of injury and the importance of examining the chemical’s packaging for product information, including chemical composition. If such information is unavailable, contacting the local poison control center, such as the American Association of Poison Control Centers at 1-800-222-1222, is advisable.

Following the history, the physical examination is the next critical step. ChatGPT’s response highlights essential components, such as conducting an initial examination to assess visual acuity and inspect for signs of conjunctival injection, corneal opacity, and epithelial defects. It also emphasizes the importance of fluorescein staining to detect epithelial damage, assessing pH levels to ensure they are within the physiologic range (not less than 7 or greater than 7.5, which may indicate ongoing chemical exposure requiring further irrigation), and performing a slit lamp examination. Additionally, intraocular pressure measurement and systemic evaluation for potential involvement are mentioned. For severe injuries, referral for further testing is recommended. However, the response overlooks the use of established classification schemes, such as the Roper-Hall classification and the Dua classification, which have been mentioned in *ICD-9* and *ICD-10 *guidelines to evaluate the extent and depth of the injury. Specifically, these schemes document corneal, conjunctival, and limbal involvement, which are critical for predicting the ultimate visual outcome.

For the treatment question (Figure 11), the analysis of ChatGPT’s response shows that it is partially correct. ChatGPT provides an outline of the appropriate treatment of chemical eye injury in 6 steps: immediate irrigation, seeking medical attention, eye examination, topical medication, pain management, and finally, follow-up. It does not clarify the recommended grading approach depending on the severity of injury, since mild burns respond well to medical treatments and lubrication, while more severe burns necessitate more intensive medical therapy and surgery. Moreover, ChatGPT’s answer missed mentioning essential components of the management, such as the preirrigation instructions, the performing technique, and the correct way to remove the foreign body.

It is thematically accurate, as it identifies the central concept and remains fully focused on the topic. The response is also coherent, with its core statement supported by the rest of the answer without contradictions. In comparison, the *ICD-9* and *ICD-10 *guidelines provide detailed clarification of each step along with detailed explanations, such as providing the types of irrigating fluids, each with different benefits. Whereas ChatGPT’s response seems to be short and brief. In addition, the *ICD-9* and *ICD-10* guidelines demonstrate that there are standard treatments and other treatments that are prescribed in special circumstances, unlike ChatGPT’s response, which categorizes them into topical medication and pain management. The probability of the need for surgery, such as debridement of necrotic epithelium, amniotic membrane disease transplantation, limbal stem cell transplant, and cultivated oral mucosal epithelial transplantation, is not mentioned in ChatGPT’s response.

For the follow-up question (Figures12  13), ChatGPT provides a detailed and structured explanation of follow-up care for chemical eye injuries. The analysis of ChatGPT’s response reveals that it is partially correct, as it accurately describes many key elements, such as reevaluation, infection monitoring, pain management, and long-term care. However, it misses critical points emphasized in the *ICD-10 *and *ICD-9* guidelines, such as the necessity of daily follow-up for severe burns and inpatient admission for compliance concerns or pediatric patients. Furthermore, while the response mentions long-term care, it fails to address specific complications like glaucoma monitoring or interventions for eyelid and conjunctival damage. The response is partially applicable. While it addresses the central concept of follow-up care and includes relevant details, it does not fully align with the thematic focus of the guidelines. Key guideline-specific elements, such as the progression of follow-up intervals, the importance of early aggressive management of complications, and the long-term need for glaucoma and dry eye monitoring, are underemphasized or omitted. The response is coherent, with its core message regarding follow-up care being consistently supported by subsequent details. The organization and flow of information are logical, providing a stepwise approach from reevaluation to long-term care. However, coherence does not compensate for the absence of critical guideline-specific details.

For the complication question (Figures14  15), ChatGPT identifies the complications of chemical eye injuries as commonly described in medical literature. The analysis of ChatGPT’s response shows that it is partially correct, providing a general overview of common complications such as corneal damage, glaucoma, and dry eye syndrome. However, certain points, such as “permanently reduced tear production” and “chronic pain,” are unnecessary or overly broad.

The response is applicable, as it focuses on the central concept of complications associated with chemical eye injuries. It lists key complications and provides brief explanations for most of them. However, the response does not fully align with the *ICD-10*/*ICD-9 *guidelines, which offer more specific details about the pathophysiology and mechanisms underlying these complications. For example, the guidelines emphasize the multifactorial causes of glaucoma (eg, inflammatory debris and damage to the trabecular meshwork) and the detailed impact of dry eye syndrome, including mucus deficiency and its effect on the tear film, which ChatGPT does not sufficiently address. The response is partially coherent. While the listed complications are thematically relevant and logically connected, the explanation lacks depth and fails to detail the progression or severity of complications.

### Quantitative Analysis

#### Overall Readability

Across all 9 sections, we found (Table 1) that the text presented an average FRES of 33.84 (SD 0.28), indicating a fairly challenging reading level. The FKGL averaged at 14.21 (SD 0.97), suggesting that the content is suitable for readers with college-level proficiency. Regarding sentence construction, the passive voice was used in 7.22% (SD 5.60%) of the sentences, implying a moderate reliance on the passive form.

**Table 1.** Overall and question-specific readability metrics (Flesch Reading Ease Score [FRES], Flesch–Kincaid Grade Level [FKGL], and passive sentence percentages) from a comparative study assessing ChatGPT-4-turbo’s responses to 9 questions on chemical ocular injuries. The artificial intelligence responses were collected on December 21, 2024, in Riyadh, Saudi Arabia.

	Sentences	Words	Syllables	FRES	FKGL	Passive sentences	Percentage of passive sentences
Section
Definition	4	92	161	35.44	14.03	0	0.00
Prevalence	14	297	500	32.15	14.86	2	14.30
Causes	10	210	367	33.12	14.47	1	10.00
Preventive measures	11	241	416	31.97	14.72	1	9.10
Symptoms	3	78	129	40.24	12.16	0	0.00
Diagnosis	19	414	716	30.12	15.24	2	10.50
Treatment	9	212	354	34.59	13.98	1	11.10
Follow-up	10	235	406	31.84	14.66	1	10.00
Complications	4	99	166	35.06	13.78	0	0.00
Mean (SD)	9.33 (5.20)	208.67 (108.66)	357.22 (199.59)	33.84 (2.97)	14.21 (0.97)	0.89 (0.78)	7.22 (5.60)

#### Category-Specific Readability Metrics

Table 2 displays the metrics across the category groupings. The mean FRES, FKGL, and passive-voice percentages for the definition, prevalence, and causes categories were 33.57 (SD 1.69), 14.45 (SD 0.42), and 8.10% (SD 7.34%), respectively. The average scores were 34.11 (SD 5.39), 14.04 (SD 1.65), and 6.53% (SD 5.70%), respectively, for the Preventive Measures, Symptoms, and Diagnosis category. In contrast, the averages were 33.83 (SD 1.74), 14.14 (SD 0.46), and 7.03% (SD 6.12%) for the treatment, follow-up, and complications categories, respectively. These findings reveal relatively low Flesch scores, indicating text complexity, with the highest average Grade Level observed in the first category (14.45). Although the percentage of passive sentences varies across categories, each remains under 10% on average.

**Table 2.** Readability metrics by category, comparison of readability metrics by grouped question categories based on ChatGPT-4-turbo responses generated during a comparative study on December 21, 2024, in Riyadh, Saudi Arabia.

Category	Sentences	Words	Syllables	FRES^a^	FKGL^b^	Passive sentences	Percentage of passive sentences
Definition, prevalence, and causes, mean (SD)	9.33 (5.03)	199.67 (102.91)	342.67 (170.76)	33.57 (1.69)	14.45 (0.42)	1.00 (1.00)	8.10 (7.34)
Preventive measures, symptoms, and diagnosis, mean (SD)	11.00 (8.00)	244.33 (168.09)	420.33 (293.58)	34.11 (5.39)	14.04 (1.65)	1.00 (1.00)	6.53 (5.70)
Treatment, follow-up, and complications, mean (SD)	7.67 (3.22)	182.00 (72.80)	308.67 (126.32)	33.83 (1.74)	14.14 (0.46)	0.67 (0.58)	7.03 (6.12)

a FRES: Flesch Reading Ease Score.

b FKGL: Flesch–Kincaid Grade Level.

#### Statistical Significance

There was no statistically significant difference in FRES (*P*=.38), FKGL (*P*=.55), or the percentage of passive sentences (*P*=.60) across the categories, as determined by one-way ANOVA. This indicates that any observed variations in readability measures were likely attributable to random fluctuations rather than systematic differences.

#### Trend Analysis

**Figure 16.**
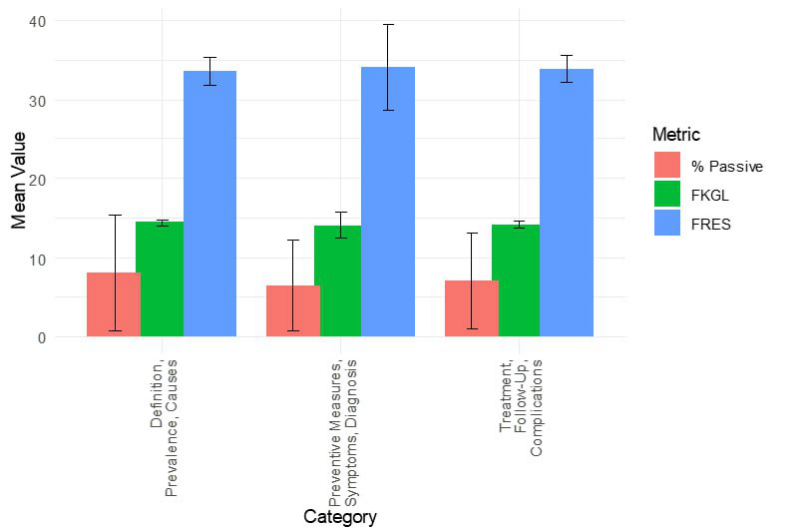
Average score for FKGL, FRES, and percentage of passive sentences across 3 question categories, based on ChatGPT-4-turbo responses about chemical eye injuries. A comparative study was conducted on December 21, 2024, in Riyadh, Saudi Arabia. FKGL: Flesch–Kincaid Grade Level;

A trend analysis showed that (Figure 16) the FRES was consistently within the low-to-mid-30s range for all categories, suggesting challenging reading material. The FKGL similarly remains around 14, underscoring the advanced reading level required to analyze the text. Minor variations in passive-voice use (ranging from 6.53% to 8.10%) indicate some stylistic differences. Overall, the readability was relatively constant across the 3 categories.

## Discussion

### Principal Findings

This study assessed ChatGPT’s performance in providing information about chemical ocular injuries. ChatGPT provided accurate responses to most queries but missed critical details such as the urgency of care and specific management protocols. The average FRES was 33.84 (± SD 0.28), and the FKGL was 14.21 (SD 0.22), indicating a high complexity level suitable for college-level readers. Statistical analysis using ANOVA showed no significant differences in readability scores across content categories (*P*=.38 for FRES, *P*=.55 for FKGL).

### Limitations

Despite its strengths, some limitations are also associated with ChatGPT. Most notably, it sometimes misses the inclusion of very important information in its responses, which is crucial for appropriate management. These issues support concerns that ChatGPT may sometimes give incomplete or biased answers, which could affect patient care in a harmful way [15]. In addition, ChatGPT cannot adjust its answers based on the user’s situation, like a patient’s history, how serious the condition is, or personal needs. This was seen in patient education, where it could not change its response depending on how simple or complex the medical case was [16].

### Comparison With Prior Work

ChatGPT responded to all queries about chemical ocular injuries. These included questions related to definition, prevalence, causes, preventive measures, symptoms, diagnosis, treatment, follow-up, and complications. The responses were mostly accurate and thematically relevant. They adhered closely to the sentence structure and grammar. Nonetheless, certain details were overlooked. They include the urgency of care in managing chemical ocular injuries and the need for exact classification systems and management protocols based on *ICD-9* and *ICD-10* guidelines. These omissions align with findings from similar studies. They highlight ChatGPT’s tendency to provide generalized but incomplete responses, reducing its clinical utility. ChatGPT accurately described the basic etiology and treatment steps but failed to include important information, such as the recommended preirrigation instructions or specific surgical interventions, which are needed in case of severe injuries. These results are consistent with those reported in another study, which evaluated the performance of ChatGPT in ophthalmology-related fields. The results showed that ChatGPT performed correctly on 46% to 58% of questions across categories. The best performance was reported in general medicine, while the poorest was reported in areas related to retina and vitreous topics. The results further support those from this study and indicate that ChatGPT provided accurate yet generalized information and often missed essential details [17].

In general, the responses were focused on the central concept of each question. There was minimal deviation from the topics. The responses related to prevalence provided statistics and global variations that were thematically relevant, although they were partially incorrect when compared with *ICD-9*/*ICD-10* standards. Thematic relevance was maintained in other topics, such as the description of diagnostic tools and complications. Nonetheless, ChatGPT introduced tangential or unrelated information, which reduced the overall coherence and thematic focus. Such a finding was also reported in prior studies that assessed the performance of AI models in medical domains [18].

The FRES and FKGL were used to assess the readability of the responses regarding chemical ocular injuries. Results showed a moderately low readability score, which suggests that the output was aligned with academic or professional standards and can limit accessibility for individuals with low levels of health literacy. The previous findings are consistent with findings from studies that evaluated the performance of ChatGPT on medical licensing examinations. The study showed that the responses were clear but overly complex for lay audiences [19]. Interestingly, there was no statistically significant difference in readability scores between the different content categories, which indicates that ChatGPT maintains a consistent language complexity regardless of the inherent difficulty. Basic information about the definition or prevalence of chemical ocular injuries was presented at the same level of complexity as more complex topics, such as treatment and surgical procedures. The same findings were reported in a meta-analysis of studies that evaluated the performance of ChatGPT in board examinations. The meta-analysis concluded that AI provided the same level of complexity across question types, irrespective of whether the questions were simple or advanced [20]. In a study assessing the performance of ChatGPT on radiology board-style examination questions, the AI demonstrated minimal variability in the complexity of responses, which indicates a uniformity in language complexity regardless of the level of difficulty of the questions [21].

While this may be beneficial to achieve uniformity, it underlines the deficiency of ChatGPT in presenting variability in speech, depending on the audience it addresses. It is desirable that simpler concepts, such as causes and symptoms, should be presented in more accessible terms, while advanced topics like surgical interventions may justifiably require technical language. This rigidity in adaptability is a finding similarly drawn from studies in other medical contexts where the responses of ChatGPT lacked subtlety in adjusting to the diversity of user groups [22]. However, readability metrics do not account for health literacy, which includes knowledge of medical terms. Even simple text can confuse a user if it includes unexplained medical jargon or lacks explanations. Besides, readability assessments do not address the accuracy of the information. This is a critical factor for patient safety and effective decision-making [19].

In this regard, ChatGPT has proven to be an exceptionally good tool for dissemination regarding chemical ocular injuries. The strong points assure that it provides structured, comprehensive answers that are logically organized, even for complex topics, in an intelligible and easy-to-understand way. Moreover, responses from ChatGPT come rather fast, allowing one to access information quickly—in some cases, this speed can be crucial in emergency situations where quick reference material is required. Recent studies, such as the work by Kianian et al [23], highlight ChatGPT’s potential to improve the accessibility of complex medical information. In their study, ChatGPT reduced the FKGL of 12 peer-reviewed uveitis papers from an average of 15th grade to 7th grade, making the content more accessible to the general public. This simplification was achieved without compromising the scientific accuracy of the information, as assessed by expert ophthalmologists [23]. Another study by Kianian et al [24] explored how ChatGPT can assist in educating patients about surgical management in glaucoma. Despite prompts aimed at generating content at a 6th-grade reading level, the resulting materials maintained a higher reading level (average 11th grade) and were deemed of “fair” quality by the DISCERN instrument. This suggests that while ChatGPT can assist in developing educational content, it may require further refinement to meet the readability needs of the average patient [24].

This is also in line with observations that ChatGPT can make information more accessible. It also leads to tailored, opportune responses, especially in medical and educational domains [25]. This also makes ChatGPT a resource for users looking for initial guidance on chemical ocular injuries, given its capability for fact retrieval and the integration of information from various topics. For instance, in medical education, ChatGPT has been noted to enhance personalized learning, improve clinical reasoning, and facilitate a better understanding of complex concepts. This is consistent with its ability to explain complex medical concepts related to ocular injuries [26].

While the complexity of language used by ChatGPT may be useful for professionals, it can be difficult for patients with lower literacy levels to understand. This mismatch in the levels of language complexity with users’ literacy is still far from optimization, according to the studies reviewed, because such an AI application could unconsciously exclude an audience that does not have a high educational background. ChatGPT’s dual nature presents both challenges and opportunities. This requires further development and control to make it more useful and reduce risks. The concept of health literacy has to be taken into consideration while educating the masses about chemical ocular injuries. Studies show that only a small percentage of adults possess proficient health literacy. This makes communicating complex medical information a significant challenge [25]. Most users struggle to read and comprehend complex medical terminology or instructions for treatment or procedures. Our findings indicate the need for ChatGPT’s content to be as readable as possible.

Future versions of AI-based platforms may adjust language complexity based on user comprehension. This could be achieved by incorporating features such as preliminary assessments or interactive dialogue to gauge the user’s understanding and tailor responses accordingly [26]. Additionally, integrating visual aids, such as step-by-step infographics or flowcharts, could enhance comprehension, particularly for inherently complex topics like chemical burn management and surgical interventions [16]. However, this study has certain limitations. Data collection was performed at one point in time within this dynamic field of AI. Furthermore, as in any qualitative research study, investigator bias might occur. In addition, the wording of the input questions can introduce some influence on the results. Future studies can overcome some of these limitations by comparing ChatGPT with other models to place it within a broader perspective in terms of its strengths and weaknesses. Other studies may wish to examine the extent to which ChatGPT is consistent and reliable across successive responses and even when asked the same question in different ways [15,27]. This might allow some insights into aspects of the capability of AI platforms to act as a reliable tool in both medical and patient education.

### Conclusions

ChatGPT shows significant potential in providing information about chemical ocular injuries, offering factually accurate and relevant responses that are generally clear and well-constructed. However, its language complexity may sometimes make information less accessible to individuals with lower health literacy. Additionally, the responses sometimes lack key information relevant to treatment, such as the urgency when dealing with chemical burns. Although ChatGPT can serve as a valuable supplementary tool for patients and health care professionals, it should not replace professional medical advice, as some responses may not align with clinical practice or address the needs of patients with different levels of education. Future iterations should aim to enhance readability, improve context-specific accuracy, and better tailor responses to user needs and literacy levels. This highlights the importance of ensuring AI tools like ChatGPT provide safe and accurate responses to support patient care and reduce the risk of misinformation.

## Abbreviations

AI: artificial intelligence
FKGL: Flesch–Kincaid Grade Level
FRES: Flesch Reading Ease Score
ICD: International Classification of Diseases
